# DAR 16-II Primes Endothelial Cells for Angiogenesis Improving Bone Ingrowth in 3D-Printed BCP Scaffolds and Regeneration of Critically Sized Bone Defects

**DOI:** 10.3390/biom12111619

**Published:** 2022-11-02

**Authors:** Eman Alfayez, Lorenzo Veschini, Monica Dettin, Annj Zamuner, Massimiliano Gaetani, Anna P. Carreca, Stevo Najman, Shahram Ghanaati, Trevor Coward, Lucy Di Silvio

**Affiliations:** 1 Faculty of Dentistry, Oral Biology Department, King Abdulaziz University, Jeddah 21589, Saudi Arabia; 2 Faculty of Dentistry, Oral & Craniofacial Sciences King’s College London, London SE1 9RT, UK; 3 Department of Industrial Engineering, University of Padua, 35131 Padua, Italy; 4 Department of Civil, Environmental, and Architectural Engineering, University of Padua, 35131 Padua, Italy; 5 Fondazione Ricerca nel Mediterraneo (Ri.MED) and Department of Laboratory Medicine and Advanced Biotechnologies, Istituto di Ricovero e Cura a Carattere Scientifico-Istituto Mediterraneo per i Trapianti e Terapie ad Alta Specializzazione, 90100 Palermo, Italy; 6 Chemical Proteomics, Department of Medical Biochemistry and Biophysics, Karolinska Institutet and SciLifeLab (Science for Life Laboratory), SE-17 177 Stockholm, Sweden; 7 Faculty of Medicine, University of Niš, 18000 Niš, Serbia; 8 Department for Oral, Cranio-Maxillofacial and Facial Plastic Surgery, Medical Center of the Goethe University, 60323 Frankfurt, Germany

**Keywords:** bone regeneration, tissue engineering, osteogenesis, angiogenesis, self-assembly peptides, biphasic calcium phosphate, 3D printing, bone scaffolds, scaffold functionalization

## Abstract

Bone is a highly vascularized tissue and relies on the angiogenesis and response of cells in the immediate environmental niche at the defect site for regeneration. Hence, the ability to control angiogenesis and cellular responses during osteogenesis has important implications in tissue-engineered strategies. Self-assembling ionic-complementary peptides have received much interest as they mimic the natural extracellular matrix. Three-dimensional (3D)-printed biphasic calcium phosphate (BCP) scaffolds coated with self-assembling DAR 16-II peptide provide a support template with the ability to recruit and enhance the adhesion of cells. In vitro studies demonstrated prompt the adhesion of both human umbilical vein endothelial cells (HUVEC) and human mesenchymal stem cells (hMSC), favoring endothelial cell activation toward an angiogenic phenotype. The SEM-EDS and protein micro bicinchoninic acid (BCA) assays demonstrated the efficacy of the coating. Whole proteomic analysis of DAR 16-II-treated HUVECs demonstrated the upregulation of proteins involved in cell adhesion (HABP2), migration (AMOTL1), cytoskeletal re-arrangement (SHC1, TMOD2), immuno-modulation (AMBP, MIF), and morphogenesis (COL4A1). In vivo studies using DAR-16-II-coated scaffolds provided an architectural template, promoting cell colonization, osteogenesis, and angiogenesis. In conclusion, DAR 16-II acts as a proactive angiogenic factor when adsorbed onto BCP scaffolds and provides a simple and effective functionalization step to facilitate the translation of tailored 3D-printed BCP scaffolds for clinical applications.

## 1. Introduction

The regeneration of critical-sized bone defects represents a significant and longstanding clinical problem [1]. Such defects result from traumas, degenerative processes, or surgery to remove cancer [2]. The current gold standard in bone regeneration remains the transplantation of autologous bone harvested from healthy regions of the skeleton [3]. Regenerating large bone defects, particularly in craniofacial applications, requires the use of implants that precisely reproduce the desired anatomical features and which have the ability to integrate with the residual bone. While autologous bone has high potential to engraft, it is difficult for surgeons to shape bone explants to match the anatomical defects. Moreover, these techniques are limited by donor site availability and morbidity [4,5,6]. Different biomaterial scaffolds have been proposed to act as bone analogues with the possibility of customization to fit the defect [7,8,9,10]. However, to date, none has achieved clinical success comparable to autologous bone transplants [11,12]. The major shortcoming of synthetic scaffolds is the difficulty of achieving adequate vascularization and cell colonization. Bone is a highly vascularized tissue where both developmental intramembranous and endochondral ossification depend on the concomitant growth of blood vessels [13,14,15]. Angiogenic blood vessels mediate the recruitment and differentiation of osteogenic precursor cells into mature osteoblasts (OB) [13,16,17]. In bone repair, the first phase of inflammation mediates the formation of the soft callus, a mesenchymal tissue that extends throughout the fracture gap connecting the ends of the fractured bone, and its invasion by newly formed blood vessels [1]. Several groups worldwide have proposed strategies for bone regeneration with cell-loaded scaffolds employing different cell types (mainly mesenchymal stem cells or MSCs) [18,19]. Although these strategies have shown some success in pre-clinical models [20], they are laborious and difficult to implement as a standard clinical treatment modality. Furthermore, there is still much debate on the cell types to be employed [21]. Hence, there is still a great need to develop more effective, clinically applicable biomaterials and scaffolds for bone tissue engineering for challenging non-self-healing defects of critical sizes. There are many reports in the literature of calcium-phosphate-based bone substitute materials for bone tissue engineering, such as hydroxyapatite (HA), tri-calcium phosphate (TCP), and biphasic calcium phosphate (BCP) [22,23,24,25]. In general, they all have demonstrable osteoconductive properties, but few have been reported to be osteoinductive per se [9]. Other materials with improved bioactivity include wollastonite and calcium doping with magnesium [26,27]. The osteoinductive potential of calcium-phosphate-based materials is highly dependent on porosity, interconnectivity, and functionalization using stimulatory proteins. 

An emerging avenue of regenerative medicine is the use of self-assembling peptides (SAPs) [28,29,30]. SAPs are unique as they can recapitulate micro-environmental niches by providing nano-topography guidance, by binding to the extracellular matrix (ECM) and by transducing mechanical forces. Synthetic ionic self-assembly oligopeptides (iSAPs) form nano structured gels in the presence of monovalent ions [31]. This nano-fibrous matrix, obtained by self-assembly, is characterized by interwoven fibres with lengths ranging from several hundred nanometres to a few microns, mimicking the extra-cellular matrix structure [32,33]. Hydrogels of self-assembling peptides comprise a large quantity of water, which represents more than the 99% of the assembled structure, thus allowing the inter-diffusion of oxygen, nutrients, and waste, as well as a bio-mimetic transport of soluble factors [34]. SAPs have several advantages, including the ability to drive differential cell responses by a specific matrix-receptor (e.g., RGD motif-binding α_v_ and β_1_ integrins) interaction [28]. In other cases, a specific peptide–receptor interaction has not been clearly identified, and it has been proposed that these peptides promote cell adhesion by presenting a favourable micro/nano-topography [35,36,37,38]. Overall, iSAPs are appealing candidates to coat and functionalize the surface of otherwise inert biomaterials [38,39]. Moreover, iSAPs can be easily enriched or covalently linked to bioactive motifs, such as growth factors [29,40] to obtain better control over kinetic release and avoid undesired side effects when trying to direct specific cell responses [41].

The rationale of this study was to use a well-described material, a 3D-printed biphasic calcium phosphate (BCP) scaffold, to demonstrate how using a simple coating method with a novel iSAP, DAR 16-II, can radically change the in vivo response of the scaffold for regenerating bone in a critical-sized bone defect. First, we demonstrated that DAR 16-II was able to promote cell adhesion and induce an angiogenic phenotype on endothelial cells (EC). Furthermore, a synergistic effect was observed on mesenchymal stem cells (MSC), whereby differentiation potential towards osteogenic precursors in vitro was enhanced. Second, the synthetic implant scaffolds manufactured by direct 3D printing of a BCP ink [42,43], and subsequently functionalized with DAR 16-II, were able to function as an architectural template, providing a favourable microenvironment for cell colonization, angiogenesis, and bone regeneration, rather than fibrous substitution, in a pre-clinical in vivo model. 

## 2. Materials and Methods

### 2.1. Preparation of DAR 16-II

DAR 16-II self-assembly oligo-peptide (ADADARARADADARAR) was synthetized and lyophilized as reported previously [38], then re-suspended in ultra-pure water at 250 µM and sterile filtered. Then, 0.5% porcine gelatin solution was prepared from gelatin powder (Sigma-Aldrich, Dorset, UK) dissolved in PBS and sterilized by autoclaving. BCP scaffolds were coated with DAR 16-II by immersion in 250 µM DAR 16-II solution in ultra-pure water for 1 h at 37 °C.

### 2.2. Fabrication of the 3D-Printed BCP Scaffold

Biphasic calcium phosphate (BCP) scaffolds containing 15% HA/85% TCP were fabricated using the direct write (DW) method described previously [42].

#### 2.2.1. Calcination and Attrition Milling

HA (product 10185602, lot 43640; Honeywell, Seelze, Germany) and β-TCP (product 21218, lot 1305078; Sigma- Aldrich, St. Louis, MO, USA) were calcined in an alumina crucible at 800 °C (for β-TCP) and 1100 °C (for HA) for 11 h. The powder was attrition milled (0.9–1.1 mm zirconia milling media; Union Process, Akron, OH, USA) in ethanol (EtOH; Fisher Scientific, Hampton, NH, USA) for 3 h (model L001, Szegvary Attritor System; Union Process). The suspension was centrifuged in an angled rotor at 8000 revolutions/min for 4 min in polycarbonate centrifuge tubes (Eppendorf AG, Hamburg, Germany). Following centrifugation, the ethanol was decanted and the solid deposit was dried in an Oakton low-temperature oven at 80 °C for 4 to 6 h. The calcined and milled ceramic powder was used for the ink formulation. Concentrated HA and β-TCP suspensions, where the volume fraction of ceramic = 0.45 to 0.5, were produced by mixing an appropriate amount of ceramic powder and ammonium polyacrylate (Darvan 821A; RT Vanderbilt, Norwalk, CT, USA) solution to disperse the particles into water. The optimal dispersant proportion per gram of ceramics was 14.5 mg, as determined by trial and error. The phases present in the sintered scaffold have been previously described [44].

#### 2.2.2. Ink Formation

The powders were added to the mixture in 2 parts: first β-TCP and then HA, according to the calculated weight, maintaining the 85:15 ratio. After each addition of powder, the suspension was mixed in the conditioning mixer (Thinky AR-250; Thinky, Tokyo, Japan) for 3 min after the addition of β-TCP, and then for 1 min once HA was added, at 2000 rpm. Next, hydroxypropyl methylcellulose (Methocel F4M, Dow Chemical Company, Midland, MI, USA) 5% weight aqueous solution was added as a thickening agent and was mixed for 1 min, then defoamed for 30 s in the conditioning mixer. As a final step, the suspension was gelled by adding poly-ethyleneimine (PEI, Product 195444, INC Biomedicals Inc, Aurora, OH, USA) 10% weight solution, and mixed and de-foamed for 1 min and 30 s, respectively.

This resulted in the final ink that was used for printing.

#### 2.2.3. Scaffold Robocasting

Gantry robotic control (Aerotech Inc., Pittsburg, PA, USA) was used to extrude colloidal ink through fine nozzles. The printing operation was both motion- and flow-rate controlled in a 3-axis motion by the custom-designed, computer-aided program RoboCAD (Robocad 3.1, 3D Inks, Stillwater, OK, USA). Square-shaped scaffolds (10 × 10-mm, 3 mm thickness with 400μm-square pores) were designed using the CAD program (Figure 4). Once a layer was printed, the nozzle was translated up (Δz) in the z-axis and another layer was printed. This process was repeated until the entire scaffold was printed. This Δz distance is a function of the deposition nozzle diameter (D) and, for all structures fabricated in this project, a Δz of 0.79D was used. The ink was housed in a syringe with internal diameters of 150–500 μm (EFD Inc, Nordson, OH, USA) and deposited through a cylindrical nozzle with an internal diameter of 330μm to produce the required scaffolds. After sintering, the diameter of bone struts was designed to be 250 μm. The ink exits the nozzle as a continuous, rod-like filament. The layers were printed at a deposition velocity of 6 to 10 μm/s in low-viscosity paraffin oil (Ultra-Pure lamp oil, Lamplight Farms Inc., Menomonee Falls, WI, USA), in order to maintain the scaffold’s shape during printing and prevent slumping of the individual layers. An alumina ceramic plate was used as the substrate (on which the scaffolds were printed) in the oil medium. 

#### 2.2.4. Heat Treating the Scaffolds

Heat treatment was carried out to sinter the scaffold material and to enhance mechanical strength. It is a slow process that takes almost 7 h with a gradual increase in temperature. The firing schedule started by heating at 4 °C/min until it reached 400 °C; the temperature was held at 400 °C for 1 h, then heated rapidly by 5 °C/min until it reached 1100 °C. This temperature was maintained for 4 h; finally, it was cooled by 9 °C/ min until the samples reached room temperature, and the desired scaffolds were achieved (Figure 4). All test BCP samples were sterilised by γ-irradiation at 1.2 Mrad before being used in the cell culture, according to the ISO 10993-5 guidelines.

### 2.3. Bioassays

#### 2.3.1. Cell Culture

Human umbilical vein ECs (HUVEC; Promocell, Heidelberg, Germany) were maintained in endothelial cell growth medium-1 (EGM-1) (Promocell, Heidelberg, Germany). Human mesenchymal stem-cell-derived mesenspheres (hMSC) were generated from primary human MSC (PromoCell, Heidelberg, Germany) with modifications of the published protocols [45]. The cells were cultured in a chemically defined DXF Medium, (PromoCell, Heidelberg, Germany). Cells were seeded at low density < 1000 cells per cm^2^ and cultured for 1 week to induce hMSC formation. Half media change was carried out every two days to maintain the spheres. Osteogenic differentiation was induced by supplementing the DXF medium with 0.1 µM dexamethasone (Dex), 0.05 mM ascorbic acid, and 10 mM β-glycerophosphate (Sigma-Aldrich, Dorset, UK). For all in vitro experiments, cells were cultured on tissue culture plastic or glass coverslips conditioned by O/N incubation with 0.5% gelatin or a solution of 0.5% gelatin containing 250 µM DAR 16-II.

#### 2.3.2. Endothelial Cell Spreading

For the cell spreading, 2 *×* 10^3^ HUVEC/well were seeded on 24-well plates in EGM-1 (Promocells, Heidelberg, Germany). Afterwards, 30’ cells were fixed with 2% buffered paraformaldehyde solution (PFA, FD Neurotechnologies, INC, Columbia, MD, USA) and permeabilized with 0.1% Triton X-100 (Sigma-Aldrich, Dorset, UK). The actinic cytoskeleton was stained with TRITC-conjugated Phalloidin (Molecular Probes, Eugene, OR, USA); nuclei were counterstained using Hoechst 33342 (ThermoFisher Scientific, Paisley, UK). Cells were imaged with a wide field inverted microscope using Cell Sens software (Olympus IX51, Biosystems, Munich, Germany). The surface area of attached cells was automatically measured using ImageJ software (with manual thresholding and then the particle analyses tool).

#### 2.3.3. Endothelial Cell Morphology

Coverslips were seeded with 3 × 10^4^ HUVEC per slide and cultured for 48 h. Cells were then fixed and immuno-stained with an anti-Vascular Endothelial Cadherin antibody (R&D Systems) followed by appropriate Alexa-488-conjugated antibody (Molecular probes). Cells were also counterstained with TRITC-conjugated phalloidin and Hoechst (Sigma-Aldrich, Dorset, UK) and imaged as before. Cells that possessed filopodia or lamellipodia were counted as “activated”, while cells without these structures and those that demonstrated peripheral distribution of the actinic cytoskeleton were counted as “quiescent” using the Cell Sense software counting tool (manual, computer-aided counting). The ratio between “activated” and “quiescent” HUVEC for each treatment was calculated and represented in a Log_10_ scale.

#### 2.3.4. Matrigel Assay

For this assay, 96-well plates were coated with 1:1 Matrigel/EGM2 (Corning, The Netherlands, Promocell, Heidelberg, Germany), with or without 250 µM DAR 16-II. Then, 1 × 10^4^ HUVEC/well in EGM-2 were seeded for each well. After 24 h, tubule-like structures were imaged as before, and measured with the cell Sense measurement tool.

#### 2.3.5. Proliferation, MTT Assay

HUVEC were seeded at a density of 5 × 10^3^ cells per well in 96-well plates coated with decreasing concentrations of DAR 16-II and cultured for 24 h in the EGM-1 medium. The MTT assay was performed according to the manufacturer’s instructions.

#### 2.3.6. hMSC Differentiation Assay

For this assay, 300 hMSC/well were cultured in the presence or absence of DAR 16-II in the presence or absence of osteogenic factors (ascorbic acid, beta-glycero-phosphate, and dexamethasone, as previously reported) in 24-well plates for 14 days. Osteogenic differentiation was evaluated using the Alizarin red stain [46] and quantified as a percentage of Alizarin-red-positive spheres over the total.

#### 2.3.7. HMSC/HUVEC Co-Culture

Here, 300 hMSC were seeded with or without 5 × 10^3^ HUVEC/well and cultured in the presence or absence of osteogenic factors in 24-well plates. After 14 days, cells were fixed with 2% PFA, permeabilized with 0.1% Triton X100, and immuno-stained with the anti-RUNX2 antibody, followed by an appropriate Alexa 488 conjugated antibody. Cells were imaged as before, and the percentage of RUNX2^+^ per sphere were quantified.

#### 2.3.8. RT^2^ Profiler PCR Array

Pellets of HUVEC were cultured in the presence or absence of DAR 16-II as in previous experiments. Total RNA was isolated using the Tri reagent (Invitrogen, Waltham, MA, USA) according to the manufacturer’s instructions. cDNA was prepared by retro-transcribing 1 µg of total RNA using the RT^2^ qPCR Array First Strand Kit (Qiagen, Crawley, UK) according to the manufacturer’s instructions. The template was mixed with RT^2^ SYBR Green/Fluorescein PCR master mix (Qiagen). Then, 10 µL were added to each well of the RT2 qPCR profiler plate containing SYBR green-optimized primer assays for 84 genes related to angiogenesis (PAHS-024Z, Qiagen, UK). The data were obtained using a Bio-Rad CFX384 analytical thermal cycler (Bio-Rad Laboratories, Hercules, HQ, USA). Threshold cycle values were analysed using the ΔΔCt method and according to manufacturer instructions for the RT2 Profiler PCR Array Kit (Cat No.: 330231, Quiagen, UK).

#### 2.3.9. Whole Proteome Analysis

Bottom-up proteomics on whole cellular protein extracts and the corresponding peptide digests was performed according to established and standardized procedures at Fondazione Ri.MED (Palermo, Italy), as previously published [47,48]. Proteomics was carried out using pellets of HUVEC (1 × 10^6^ cells/ sample) cultured for 48 h in the presence or absence of DAR 16-II, in triplicate and similarly to previous experiments. After in-solution trypsin digestion, DAR-16-II-treated and control triplicate samples were labelled with reporter ions for tandem mass tag (TMT) isobaric labelling technology [49] using TMTsixplex reagents (Thermo Fisher Scientific) according to the manufacturer’s instructions. The resulting sample was fractionated using a high-pH reversed-phase peptide fractionation kit in two replicate fraction series (Pierce, Thermo Fisher Scientific), as previously described [47]. Each fraction was analysed by Nanoscale liquid chromatography coupled with tandem mass spectrometry (nLC- MS/MS) using a Q-Exactive mass spectrometer (Thermo Fisher Scientific) equipped with a Nanospray Flex ion source and coupled to an UHPLC Ultimate 3000 system (Dionex, Thermo Fisher Scientific), which was configured with on-line sample pre-concentration and desalting; a resolution of 70,000 was maintained in both full MS and MS/MS modes. *Protein identification, quantification, and data analysis:* raw data of both series of sample fractions were analysed using the Proteome Discoverer 2.1 software (PD2.1, Thermo Fisher Scientific). Data were organized in one single study including both replicate fraction series. MS/MS peptide spectrum matching was performed using combined Sequest HT and Mascot search engines against the SwissProt *Homo sapiens* full proteome database (taxonomy identification number 9606), setting fragment mass tolerance as 0.05 Da, the minimum peptide length as 6 amino acids, a maximum of two trypsin MIS cleavages, and a threshold of false discovery rate (FDR) of 0.01% for highly confident identification. Only master proteins were selected, to avoid redundancy. Relative peptide and protein quantification of sample replicates from DAR-16-II-treated HUVEC cultures—labelled with TMT^6^-126, TMT^6^-127 and TMT^6^-128 reporter ions—was performed relative to the abundance of one control sample (labelled with the TMT^6^-130 reporter ion) while the abundance values of the other two control samples (labelled with the TMT^6^-129 and TMT^6^-131 reporter ions), were used to measure the intrinsic variability of each protein amount in HUVEC. Their average ratios over TMT^6^-130 and their average deviation from it were protein selection criteria, excluding proteins with control/control ratio outranging values between 0.67 and 1.5, and with deviation more than 0.5. Only unique and top three peptides were used for protein quantification. The identified proteins with a percentage change of the protein quantification ratio of less than 40% were also filtered out. Functional data analysis was then performed by looking at gene ontology biological processes, accessing protein databases through the Protein Center Annotation tool of PD2.1. 

#### 2.3.10. In Vivo Study

The study was performed in collaboration with the Faculty of Medicine, University of Niš, Serbia, with the approval of the Local Ethical Committee. Four rabbits (*n* = 4) were randomly distributed into the study groups. In each rabbit, three critical-size bone defects (10 mm in diameter) were created (one central defect in the midline of frontal squama of the frontale and one defect in each parietal bone; two defects in both sides of the parietal bone). Two were implanted with BCP scaffolds with and without DAR 16-II, and one was left empty as a control. Half of the BCP scaffolds were coated with DAR 16-II by immersion in 250 µM DAR 16-II solution in ultra-pure water for 1 h at 37°C to allow DAR 16-II assembly on the surface of the BCP. After the placement of BCP scaffolds into the two parietal bonet defects, the incision was sutured in layers. After 8 weeks, the rabbits were euthanized by one overdose of ketamine and the calvaria bones were harvested for radiographic and histological examination. Following excision, the explants were fixed in 10% neutral-buffered formalin for 48 h and decalcified in 10% ethylene-diamine-tetraacetic (EDTA) acid at room temperature for 7–10 days. Specimens were dehydrated in a series of increasing alcohol concentrations, followed by Xylol treatment and embedding in paraffin. Then, 3–5 µm slices were stained with Haematoxylin and Eosin (H&E) and Azan stains, according to standard methods. Slides were visualized and imaged with a Zeiss light microscope (Zeiss PrimoStar HD, Carl Zeiss Microscopy GmbH, Jena, Germany). Histo-morphological quantification was performed on 100× OM images using ImageJ.

### 2.4. SEM and EDS Coupled SEM

SEM and SEM-EDS were performed at the Physics Photonic Laboratory at the department of Physics (King’s College London). Standard 22 × 22 mm square coverslips (VWR, Radnor, PA, USA) were coated either with gelatin or gelatin:DAR 16-II (1:1) in 6-well plates. BCP scaffolds were immersed in 300 µL of 250 µM DAR16-II solution for 1 h at 37 °C prior to imaging. Non-coated scaffolds were used as controls. Three scaffolds from each group were selected. Scanning electron microscopy (SEM) samples were dry mounted on stubs using carbon tape, and subsequently coated with a 10 nm gold layer in a QR150R sputter system, while Energy Dispersive Spectrometry (EDS) samples were coated with 10 nm carbon (Quorum Technologies, East Sussex, UK). Images and elemental analysis were performed using a SEMFEG Hitachi S4000 Scanning Electron Microscope with an Oxford Instrument INCA EDS system (Vext = 20 keV; i = μA; Hitachi, Tokyo, Japan).

### 2.5. Micro Bicinchoninic Acid (BCA) Assay

BCP scaffolds were immersed in 300 µL of 250 µM DAR16-II solution for 1 h, followed by immersion in 0.5 mL PBS (Sigma-Aldrich, Dorset, UK) for 1 h at 37 °C. The protein content in the PBS was measured using a QuantiPro BCA Assay Kit (SigmaAldrich, Dorset, UK). Fresh PBS was used as a control. The fluorescence was measured using a fluorometric plate reader (ChameleonTM, Hidex, Finland) 620 nm. The protein content was calculated from the standard curve.

### 2.6. Statistical Analysis

Results are expressed as the mean ± standard deviation (SD). Data were analysed using either one-way or two-way ANOVA with Tukey’s Multiple Comparison post hoc to compare the means among groups. GraphPad Prism 6.0c software (GraphPad Software Inc., La Jolla, CA, USA) was used as the statistical software. Significance was predetermined at α = 0.05. Statistical differences were designated as significant if *p*-values were less than 0.05 (* *p* ≤ 0.05), and as highly significant if *p*-values were less than 0.01 (** *p* ≤ 0.01) or less than 0.001 (*** *p* ≤ 0.001). 

## 3. Results

### 3.1. DAR 16-II Drives Morphological Changes in Endothelial Cells, Reminiscent of Angiogenic Activation

**Figure 1:**
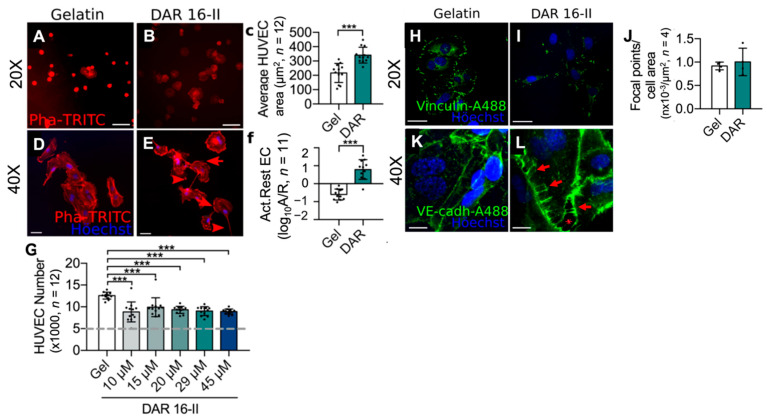
HUVEC are activated on exposure to DAR 16-II. (**A**,**B**) Representative images (Pha staining, 20× OM, scale-bars= 30 μm) of HUVEC cultured in the presence or absence of DAR 16-II at 30 min post seeding; (**C**) evaluation of cells’ average area; (**D**,**E**) representative images (Pha Staining, 40× OM, scale-bars = 30 μm) of HUVEC grown on gelatin or DAR 16-II. Dar-16-II induces morphological changes, i.e., the presence of filopodia (red arrow) and lamellipodia (red arrowhead) is suggestive of an activated, migratory phenotype; (**F**) quantification of the ratio between “activated” and “quiescent” HUVEC; (**G**) proliferation of HUVEC (24 h) when cultured in different concentrations of DAR 16-II or gelatin as control (the grey dashed line indicates the input number of HUVEC = 5 × 10^3^); (**H**,**I**) representative pictures (Vinculin immuno-staining, 20× OM, scale-bars = 30 μm) demonstrating the re-distribution of focal adhesions in HUVEC seeded in the presence of DAR16-II when compared to the control; (**J**) quantification of vinculin-positive foci per cell area; (**K**,**L**) representative images (VE-Cadherin immuno-staining, 40× OM, scale-bars = 10 μm) showing inter-endothelial junctions of HUVEC monolayers grown on DAR 16-II or gelatin. HUVEC cultured on DAR 16-II display interdigitating junctions (red arrows) with discontinuities (red asterisk). *** *p* < 0.05.

To investigate the angiogenic potential of DAR 16-II, human umbilical vein endothelial cells (HUVEC) were cultured in the presence or absence of DAR 16-II. HUVEC seeded in the presence of DAR 16-II rapidly adhered (30’) and spread faster than on gelatin (used as a control), as demonstrated by TRITC-labelled phalloidin (Pha, to visualize actin cytoskeleton) staining and cell area quantification (Figure 1A–C).

HUVEC exposed to activating/migratory stimuli (e.g., VEGF) are known to acquire an elongated morphology (spindle shape) when compared to quiescent ones (polygonal), displaying features such as filopodia and lamellipodia (e.g., Figure 1D,E). We observed that culturing cells on DAR 16-II induced HUVEC to acquire an activated-migratory phenotype with cytoskeletal remodelling when compared to gelatin, as demonstrated by morphological analysis after 48h culture and upon TRITC-Pha staining (Figure 1D–F). Proliferation assays demonstrated that culture on DAR 16-II inhibited but did not completely abolish HUVEC proliferation (by approximately 30%, Figure 1G). Focal adhesion staining (FA, Vinculin) of HUVEC on DAR 16-II demonstrated a re-distribution of FAs (Figure 1H,I), but the total number of Vinculin-positive foci per cell area was not changed (Figure 1J), suggesting that DAR 16-II does not influence Vinculin expression. VE-Cadherin staining demonstrated that HUVEC cultured on DAR 16-II resulted in looser inter-interdigitating rather than linear, inter-endothelial junctions with the presence of discontinuities in the monolayer (Figure 1K,L) [50]. Taken together, these findings suggest the induction of the EC phenotype resembles the morphology and characteristics of migrating angiogenic tip cells [51] upon exposure to DAR 16-II. Angiogenesis is characterized by distinct phenotypic changes in vascular endothelial cells; as a result of our findings, we further investigated the effects of DAR 16-II on morphogenesis.

### 3.2. DAR 16-II Switches on a Migratory and Morphogenetic Program in EC

**Figure 2:**
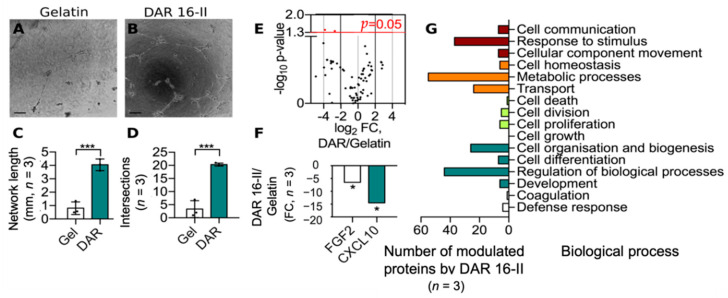
(**A**,**B**) Representative images (10× OM, scale-bars = 100 mm) of tubule-like structures formed by HUVEC cultured on Matrigel or DAR-16-II-enriched Matrigel; (**C**,**D**) the quantification of tubule-like structures’ lengths and interconnectivity; (**E**) a volcano plot representation of the datasets obtained from a low-density qRT-PCR-based array. Red dots represent differentially expressed genes; (**F**) a fold change representation of two down-regulated genes (*p* < 0.05. FGF2, CXCL10) from the qRT-PCR-based array dataset; (**G**) GO analysis showing the number of differentially regulated protein amounts identified and quantified by mass-spectrometry-based proteome analysis and corresponding enriched processes. * *p* < 0.05, *** *p* < 0.001.

HUVEC cultured on Matrigel forms tubule-like structures resembling pre-vascular networks [52]. Culturing HUVEC on DAR 16-II-enriched Matrigel resulted in the formation of longer and more complex networks of tubule-like structures, compared to Matrigel alone (Figure 2A–D), suggesting that DAR 16-II imparts an angiogenic phenotype on HUVEC. To characterize the molecular signature induced by DAR 16-II in HUVEC, an established panel of 84 key mediators of angiogenesis were measured by a qRT-PCR based array (Quiagen RT^2^ Profiler).

The results showed the down-regulation of FGF2 and CXCL-10 (−6.5 folds and −14.5 folds, respectively, Figure 2E,F), among the assayed regulators of angiogenesis. Furthermore, whole proteome analysis highlighted substantial changes in the proteome of HUVEC cultured on DAR 16-II (Figure 2G, Tables S1 and S2). In particular, proteins involved in cell adhesion (e.g., HABP2), migration (e.g., AMOTL1), cytoskeletal re-arrangement (e.g., SHC1, TMOD2), immuno-modulation (e.g., AMBP, MIF), and morphogenesis (e.g., COL4A1) were found to be up-regulated, while negative modulators of these functions (e.g., PFN3, SHROOM2) were found to be down-regulated. Gene ontology (GO) analysis demonstrated the enrichment of biological processes involved in response to stimuli, metabolism, and morphogenesis (Figure 2G), supporting our previous functional observations. Overall, the data on HUVEC strongly suggested that exposure to DAR 16-II imparted an angiogenic phenotype to ECs.

### 3.3. DAR-16-II-Activated Endothelial Cells Promote MSC Differentiation

**Figure 3:**
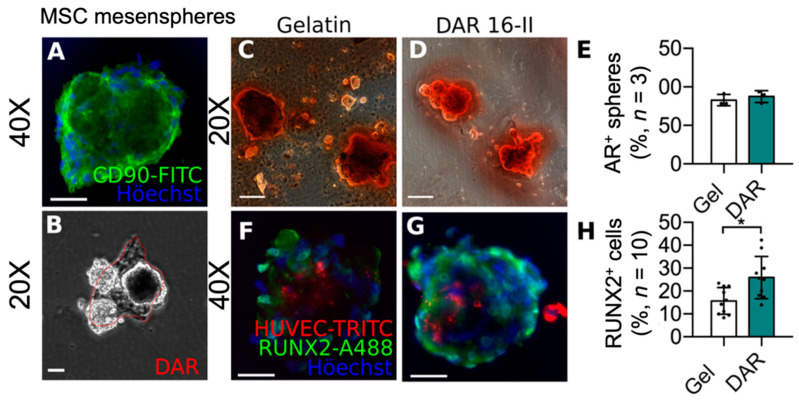
(**A**) Expression of the mesenchymal stem cell marker CD90 on freshly generated mesenspheres (40× OM, scale-bar = 100 μm); (**B**) representative picture (20× OM, scale-bar = 100 μm) showing mesenspheres’ adhesion to DAR 16-II; (**C**,**D**) representative pictures (20× OM, scale-bar = 100 μm) of Alizarin red-stained mesenspheres grown under osteogenic conditions; (**E**) average number of alizarin positive spheres (average of three independent experiments counting twenty spheres for each experiment); (**F,G**) representative pictures (40× OM, scale-bars = 100 μm) of RUNX2 immuno-stained mesenspheres; (**H**) percentage of RUNX+ cells/mesensphere (average of ten spheres from three independent experiments). * *p* < 0.05.

The differentiation of mesenchyme-derived osteogenic precursors is paramount in bone regeneration, and is associated with angiogenesis in vivo. Hence, we sought to investigate the potential effects of DAR16-II on the early differentiation of osteogenic precursors. To this end, we generated human mesenchymal stem cells (hMSC)-derived mesenspheres [45] and cultured them on DAR 16-II or gelatin (Figure 3A–D) in the presence or absence of osteoinductive factors (OF) (Figure 3C,D). Mesenspheres that did not adhere to cell culture plastic under standard conditions promptly adhered to DAR 16-II but not to gelatin (Figure 3B).

After 14 days, the cultures were assayed for the deposition of mineralized matrix (Alizarin red stain, AR) as an index of hMSC osteogenic differentiation. None of the hMSCs cultured in the absence of OF were found to be AR positive (data not shown) while most of those grown in the presence of OF were AR positive independently from the culture substrate (Figure 3C–E). To further investigate the potential effects of DAR-16-II on hMSC differentiation, immunostaining for the early marker of osteogenic differentiation, RUNX2, was performed. As in previous experiments, no differences were observed in hMSC cultured on DAR 16-II or gelatin (data not shown). These data suggest that DAR 16-II does not affect the osteogenic differentiation of MSC per se. Since active angiogenesis and osteogenesis are coupled in vivo [13,17], we sought to determine whether the observed activation of HUVEC upon exposition to DAR 16-II could enhance the differentiation of hMSC into osteogenic cells. To this aim, mesenspheres were cultured in the presence or absence of DAR 16-II and RFP-tagged HUVEC under osteogenic conditions. Immunostaining for RUNX2 after 10d co-culture (Figure 3F,G) demonstrated a higher percentage of RUNX2^+^ cells within hMSC cultured on DAR 16-II in comparison to gelatin (Figure 3H). Each sphere was found to adhere onto two to three HUVECs in both experimental cases (data not shown); thus, we concluded that the observed enhanced differentiation of hMSC could be mediated by differential “angiocrine” signalling from DAR-16-II-activated HUVEC. In this sense, it has been shown that FGF2 inhibits MSC differentiation into osteogenic precursors, while HUVEC cultured on DAR-16 II downregulated this factor (Figure 2F). It can be speculated that, in our experimental system, DAR-16-II-activated HUVEC could favour the osteogenic differentiation of hMSC by relieving them of their FGF2-mediated inhibition, but this hypothesis warrants more thorough investigation. Overall, our in vitro data demonstrated that DAR 16-II supports the prompt adhesion of EC- and hMSC-derived mesenspheres without exerting any cytotoxic effect, and that it promotes EC activation. These DAR-16-II-activated ECs were found to enhance hMSC differentiation toward RUNX2-expressing osteogenic precursors.

### 3.4. DAR 16-II Effectively Coats Microporous 3D-Printed Scaffolds

**Figure 4:**
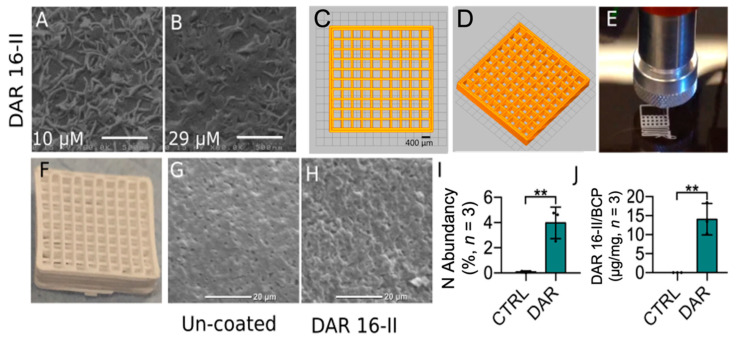
The preparation and characterization of 3D-printed BCP scaffolds enriched with DAR 16-II. (**A**,**B**) SEM imaging of DAR-16-II-coated glass slides: DAR 16-II formed nano-structures with major axes in the 250–500 nm range, and presenting finer irregularities in the 50–100 nm range; (**C**,**D**) BCP 3D-printed scaffolds with 400 μm pores; (**E**,**F**) 3D direct printing of BCP at 200 μm resolution and the final scaffold after sintering; (**G**,**H**) SEM images of un-coated or DAR-16-II-coated BCP; (**I**) elemental analysis of BCP-coated and un-coated samples (three randomly picked spots per sample) demonstrated the presence of nitrogen on the BCP surface related to the peptide coating; (**J**) quantification of DAR 16-II adsorption on the BCP scaffolds through a micro-BCA assay. ** *p* < 0.01.

To provide a proof of concept for the potential use of DAR 16-II in bone-regenerative strategies, we performed an in vivo study employing a critical size calvaria (frontal, occipital, and parietal cranial bones) defect model in rabbits. In brief, 3D-printed BCP scaffolds were designed to match the experimental defects. Scaffolds with 400 mm porosity were tested (Figure 4C,D), 3D printed at 200 mm resolution (Figure 4E,F), and coated with DAR 16-II.

To analyse the morphology of the hydrogels formed by DAR 16-II, DAR-16-II-coated glass slides were imaged by SEM. SEM imaging showed that DAR 16-II formed pellicles by overlapping irregular and relatively flat structures with major axes in the 250–500 nm range, and presenting finer irregularities in the 50–100 nm range (Figure 4A,B). The BCP scaffolds (Figure 4F) possessed a micro-porous surface topography with micro pits 1–3 mm in diameter (Figure 4G,H), which promotes cell adhesion [42]. The DAR 16-II coating of the BCP scaffolds adapted to the topography of the supporting material (Figure 4H). Elemental analysis by energy-dispersive spectroscopy (EDS)-coupled SEM on the BCP coated and uncoated samples (three randomly selected areas per sample) confirmed the presence of the peptide coating, as assessed by the presence of a peak corresponding to nitrogen (Figure 4I). These results were confirmed and quantified by a micro-BCA assay (Figure 4J).

### 3.5. DAR-16-II-Coated Scaffolds Improve Bone Regeneration and Inhibit Fibrosis

**Figure 5:**
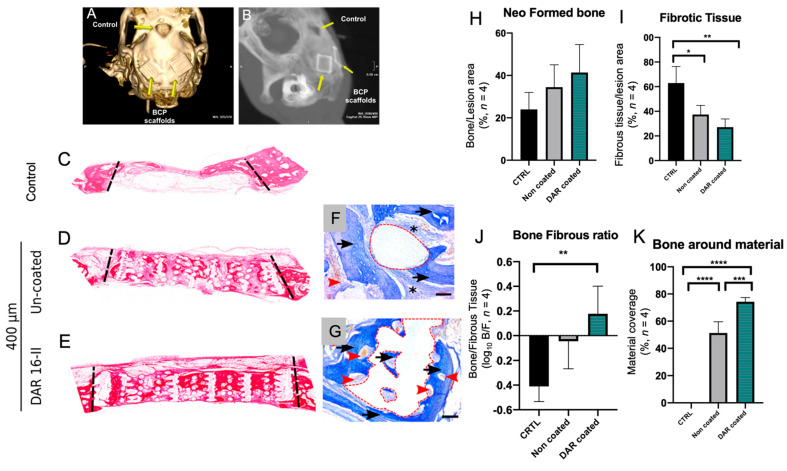
In vivo evaluation of DAR-16-II-coated 3D printed BCP scaffolds (*n* = 4). (**A**,**B**) CT scan and X-ray images of calvaria defects, either left empty as a control or implanted with BCP scaffolds (yellow arrows); (**C**) image of a section of calvaria bone defect, without an implant, used as a control; (**D**,**E**), calvaria sections of implants with 400 µm un-coated or coated with DAR 16-II; pictures are reconstructions from 100× OM images; (**F**,**G**) representative histological images (Azan stain, 100× OM, scale-bars = 100 μm) of calvaria bones implanted with 3D-printed scaffolds (red dashed line). Black arrows indicate neo-formed bone, black asterisks indicate fibrotic tissue, and red arrowheads indicate blood vessels; (**H**) quantification of the total bone formed within the lesion area; (**I**) quantification of the total fibrous substitution within the lesion area; (**J**) ratio between neo-formed bone and fibrous substitution_;_ (**K**) quantification of the material covered by the neo-bone (percentage of material surface, red dashed line, in contact with bone). * *p* < 0.05, ** *p* < 0.01, *** *p* < 0.001, **** *p* < 0.0001.

New bone generation in all implanted samples was demonstrated by the histological staining of calvaria sections with Azan and Haematoxylin and eosin (H&E) (Figure 5C–G). The total amount of regenerated bone, as measured by histomorphometry, was observed to be higher in the samples implanted with the scaffold alone as compared to no-implant controls; however, the results were not statistically significant (Figure 5I).

The ratio between newly formed bone and fibrous tissue substitution (Figure 5J) clearly demonstrated that the coated implants were able to promote favourable bone ingrowth with minimal fibrosis. Moreover, the DAR-16-II-coated implant (Figure 5G red dashed line) was mostly covered by newly formed bone (intense blue) and there was an absence of fibrotic tissue, in sharp contrast to the observations made of the uncoated sample (Figure 5F asterisks). The quantification of the material covered by newly formed bone (Figure 5K) confirmed the qualitative observations. All of the newly formed bone was viable and well vascularized, as demonstrated by the presence of micro-vessels containing red blood cells (Figure 5F,G, red arrowheads).

Overall, we concluded that DAR 16-II was able to induce qualitatively better bone repair by promoting vascularization and bone regeneration, rather than fibrous substitution.

## 4. Discussion

Conventional bone grafts have associated limitations; tissue engineering based on three-dimensional customised printed scaffolds has emerged as a promising approach to bone repair and regeneration. Ideal scaffolds for bone tissue engineering should be biocompatible, osteoconductive, osteoinductive, inhibit adverse host inflammatory and fibrotic responses, and allow tissue ingrowth within the bulk of the material and its eventual substitution with neo-formed bone. The process of reconstructing and regenerating cranial bone defects presents additional challenges due to the different ossification mechanisms of these bones (which are intramembranous rather than endochondral) and their complex anatomy. Moreover, 3D-printed porous scaffolds not only serve as a structural template for tissue regeneration, but they also provide complex signaling cues to cells and facilitate oxygen and metabolic activity. In this work, we directly printed biomaterials into 3D scaffolds, as reported previously [53]. This method of fabrication allows for the creation of scaffolds of the desired shape, size, and porosity, starting from digital models. Maxillofacial surgeons and prosthetists are increasingly implementing and employing digital 3D technologies for diagnosis (CT scans and 3D reconstruction) and for surgical/orthognathic planning [54]. Therefore, the possibility of designing and fabricating scaffolds by employing the same digital tools and source data (patients’ anatomy through CT scans) is a promising new strategy for personalized bone tissue engineering. Such 3D scaffolds are able to mimic the natural extracellular matrix, providing a structural template that is able to support cell adhesion, migration, differentiation, and proliferation, and provide guidance for neo-tissue formation. The properties of the biomaterial used (BCP) indicate that the scaffold is biocompatible and osteoconductive; it can also be rendered osteoinductive in nanocrystalline forms [55]. Non-functionalized BCP has previously been demonstrated to induce some degree of inflammation and fibrosis [56]; this was also confirmed by our in vivo data (Figure 5I,J).

Aiming to improve cellular responses in vivo, the BCP scaffold was functionalized with DAR 16-II. Angiogenesis requires coordinated changes in endothelial cell morphology and gene expression, as we demonstrated and characterized in vitro (Figure 1, Figure 2 and Figure 3). DAR 16-II induced the rapid adhesion of EC and hMSC, conferred an activated, more angiogenic phenotype to HUVEC, and sustained better hMSC differentiation toward the osteogenic lineage. Cells are influenced by their microenvironment; DAR16-II is able to mimic the physiological niche by the adsorption of other proteins favouring the recruitment and adhesion of cells, thus supporting the regulation of cellular processes such as the mediation of cell-surface receptor-integrin expression. A possible mechanism of action of DAR16-II is via the retention of angiogenic factors, such as VEGF and heparin at the site during the vessel regeneration stage [57]. Furthermore, another mechanism of cell proliferation and activation toward the pro-angiogenic phenotype, related to the Hippo signalling and transcriptional coactivator of YAP and TAZ, may be involved [50,58]. In fact, it has been demonstrated that nanofibers of self-assembling peptide RADA can influence neurogenesis through mechano-transductor signals [59]. In a similar way, DAR16-II could drive HUVEC toward a pro-angiogenic phenotype through YAP/TAZ activation due to the mechanosensing of endothelial cells (data not shown). Overall, angiogenesis and osteogenesis are coupled, and these data are consistent with the hypothesis that DAR 16-II could improve bone healing by inducing an early angiogenic response and thus prevent a fibrous reaction [60]. In our in vivo experimental model, uncoated BCP induced a mixed fibrotic/osteogenic response at eight weeks post implantation—the time at which most of the reparatory processes are terminated. In contrast, in the DAR-16-II coated scaffolds, most of biomaterial surface was covered with neo-formed bone (Figure 5H–K). Porosity and mesh architecture are primarily involved in favouring (or impeding) the adsorption of blood proteins (Vroman effect) and the inflow/migration of cells within the scaffold, which are paramount processes in initial scaffold integration. Thus, iSAPs (DAR 16-II in particular) are a promising route for bone tissue engineering, particularly in the reconstruction of large bone defects where the local cellular environment is usually compromised. Wound healing is a well-orchestrated process; however, in cases where the defect is large, the healing response can become chronic or dysregulated and this can lead to the development of pathological fibrosis. In these cases, we provide a viable strategy to tailor and functionalize the scaffolds to achieve maximal cell colonization and minimize fibrosis. Furthermore, the applicability of this strategy can be extended to the regeneration of other tissues and organs, and for drug delivery that incorporates active peptide sequences from the desired proteins. Therefore, allowing for the controlled placement of specific binding domains on scaffolds induces and facilitates cellular responses in a temporal and selective manner [61].

## 5. Conclusions

A major challenge to bring engineered scaffolds into the clinical setting is the ease with which the manufacturing strategies can be translated into GMP standards. The strategy here employed was based on biocompatible synthetic materials that have the flexibility to be manufactured in a customized manner to a desired shape and size, hence facilitating their potential to be readily translated into a standardized ‘off-the shelf’ product. The present study demonstrated a simple and viable strategy for coating and functionalizing 3D-printed BCP scaffolds. Immersion of the scaffold in a DAR 16-II solution conveniently provided a stable coating. The angiogenic potential of DAR 16-II was confirmed, and osteogenic induction was observed via the activation of endothelial cells. Overall, the present study demonstrated a viable strategy for the fabrication of mechanically stable, osteoconductive, and osteoinductive scaffolds for the regeneration of critical-sized bone defects. These exciting findings warrant further mechanistic studies to determine the exact mode of action of DAR 16-II in promoting favourable angiogenic/osteogenic and inflammatory responses, thus sustaining a reparative rather than substitutive fibrotic process.

## Data Availability

Raw data to reproduce the described findings are available upon request. The raw mass spectrometry data will be made available through a public repository; however, they cannot be shared at this time due to time limitations.

## Supplementary Materials

The following supporting information can be downloaded at: https://www.mdpi.com/article/10.3390/biom12111619/s1, Table S1: List of up-regulated proteins; Table S2: List of down-regulated proteins.

## References

[1] Loi F., Córdova L.A., Pajarinen J., Lin T., Yao Z., Goodman S.B. (2016). Inflammation, Fracture and Bone Repair. Bone.

[2] Su N., Villicana C., Yang F. (2022). Immunomodulatory Strategies for Bone Regeneration: A Review from the Perspective of Disease Types. Biomaterials.

[3] Vidal L., Kampleitner C., Brennan M.Á., Hoornaert A., Layrolle P. (2020). Reconstruction of Large Skeletal Defects: Current Clinical Therapeutic Strategies and Future Directions Using 3D Printing. Front. Bioeng. Biotechnol..

[4] Janssen N.G., Weijs W.L.J., Koole R., Rosenberg A.J.W.P., Meijer G.J. (2014). Tissue Engineering Strategies for Alveolar Cleft Reconstruction: A Systematic Review of the Literature. Clin. Oral. Investig..

[5] Zizzari V.L., Zara S., Tetè G., Vinci R., Gherlone E., Cataldi A. (2016). Biologic and Clinical Aspects of Integration of Different Bone Substitutes in Oral Surgery: A Literature Review. Oral Surg. Oral Med. Oral Pathol. Oral Radiol..

[6] Fishero B., Kohli N., Das A., Christophel J., Cui Q. (2015). Current Concepts of Bone Tissue Engineering for Craniofacial Bone Defect Repair. Craniomaxillofacial Trauma Reconstr..

[7] Idowu B., Cama G., Deb S., Di Silvio L. (2014). In Vitro Osteoinductive Potential of Porous Monetite for Bone Tissue Engineering. J. Tissue Eng..

[8] Wang M.O., Vorwald C.E., Dreher M.L., Mott E.J., Cheng M.-H., Cinar A., Mehdizadeh H., Somo S., Dean D., Brey E.M. (2015). Evaluating 3D-Printed Biomaterials as Scaffolds for Vascularized Bone Tissue Engineering. Adv. Mater..

[9] El-Ghannam A. (2005). Bone Reconstruction: From Bioceramics to Tissue Engineering. Expert Rev. Med. Devices.

[10] Yazdanpanah Z., Johnston J.D., Cooper D.M.L., Chen X. (2022). 3D Bioprinted Scaffolds for Bone Tissue Engineering: State-Of-The-Art and Emerging Technologies. Front. Bioeng. Biotechnol..

[11] Dimitriou R., Jones E., McGonagle D., Giannoudis P.V. (2011). Bone Regeneration: Current Concepts and Future Directions. BMC Med..

[12] Bhumiratana S., Bernhard J.C., Alfi D.M., Yeager K., Eton R.E., Bova J., Shah F., Gimble J.M., Lopez M.J., Eisig S.B. (2016). Tissue-Engineered Autologous Grafts for Facial Bone Reconstruction. Sci. Transl. Med..

[13] Maes C., Kobayashi T., Selig M.K., Torrekens S., Roth S.I., Mackem S., Carmeliet G., Kronenberg H.M. (2010). Osteoblast Precursors, but Not Mature Osteoblasts, Move into Developing and Fractured Bones along with Invading Blood Vessels. Dev. Cell.

[14] Clarkin C., Olsen B.R. (2010). On Bone-Forming Cells and Blood Vessels in Bone Development. Cell Metab..

[15] Zhao H., Feng J., Ho T.-V., Grimes W., Urata M., Chai Y. (2015). The Suture Provides a Niche for Mesenchymal Stem Cells of Craniofacial Bones. Nat. Cell Biol..

[16] Maes C., Goossens S., Bartunkova S., Drogat B., Coenegrachts L., Stockmans I., Moermans K., Nyabi O., Haigh K., Naessens M. (2010). Increased Skeletal VEGF Enhances β-Catenin Activity and Results in Excessively Ossified Bones. EMBO J..

[17] Kusumbe A.P., Ramasamy S.K., Adams R.H. (2014). Coupling of Angiogenesis and Osteogenesis by a Specific Vessel Subtype in Bone. Nature.

[18] Péault B., Asatrian G., Pham D., Hardy W.R., James A.W. (2015). Stem Cell Technology for Bone Regeneration: Current Status and Potential Applications. SCCAA.

[19] Sui B.-D., Hu C.-H., Liu A.-Q., Zheng C.-X., Xuan K., Jin Y. (2019). Stem Cell-Based Bone Regeneration in Diseased Microenvironments: Challenges and Solutions. Biomaterials.

[20] Yu H.-S., Won J.-E., Jin G.-Z., Kim H.-W. (2012). Construction of Mesenchymal Stem Cell–Containing Collagen Gel with a Macrochanneled Polycaprolactone Scaffold and the Flow Perfusion Culturing for Bone Tissue Engineering. BioRes. Open Access.

[21] Colnot C. (2011). Cell Sources for Bone Tissue Engineering: Insights from Basic Science. Tissue Eng. Part B Rev..

[22] Baino F., Novajra G., Vitale-Brovarone C. (2015). Bioceramics and Scaffolds: A Winning Combination for Tissue Engineering. Front. Bioeng. Biotechnol..

[23] Islam M.T., Felfel R.M., Abou Neel E.A., Grant D.M., Ahmed I., Hossain K.M.Z. (2017). Bioactive Calcium Phosphate–Based Glasses and Ceramics and Their Biomedical Applications: A Review. J. Tissue Eng..

[24] Rao S.H., Harini B., Shadamarshan R.P.K., Balagangadharan K., Selvamurugan N. (2018). Natural and Synthetic Polymers/Bioceramics/Bioactive Compounds-Mediated Cell Signalling in Bone Tissue Engineering. Int. J. Biol. Macromol..

[25] Buranawat B., Di Silvio L., Deb S., Nannmark U., Sennerby L., Palmer R.M. (2014). Evaluation of a β-Calcium Metaphosphate Bone Graft Containing Bone Morphogenetic Protein-7 in Rabbit Maxillary Defects. J. Periodontol..

[26] Lin Y.-H., Lee A.K.-X., Ho C.-C., Fang M.-J., Kuo T.-Y., Shie M.-Y. (2022). The Effects of a 3D-Printed Magnesium-/Strontium-Doped Calcium Silicate Scaffold on Regulation of Bone Regeneration via Dual-Stimulation of the AKT and WNT Signaling Pathways. Biomater. Adv..

[27] Lin S., Yang G., Jiang F., Zhou M., Yin S., Tang Y., Tang T., Zhang Z., Zhang W., Jiang X. (2019). A Magnesium-Enriched 3D Culture System That Mimics the Bone Development Microenvironment for Vascularized Bone Regeneration. Adv. Sci..

[28] Peng F., Zhang W., Qiu F. (2020). Self-Assembling Peptides in Current Nanomedicine: Versatile Nanomaterials for Drug Delivery. CMC.

[29] Zhang S. (2017). Discovery and Design of Self-Assembling Peptides. Interface Focus..

[30] Najafi H., Jafari M., Farahavar G., Abolmaali S.S., Azarpira N., Borandeh S., Ravanfar R. (2021). Recent Advances in Design and Applications of Biomimetic Self-Assembled Peptide Hydrogels for Hard Tissue Regeneration. Bio-Des. Manuf..

[31] Gelain F., Luo Z., Zhang S. (2020). Self-Assembling Peptide EAK16 and RADA16 Nanofiber Scaffold Hydrogel. Chem. Rev..

[32] Damodaran S., Parkin K.L. (2017). Fennema’s Food Chemistry.

[33] Koutsopoulos S. (2016). Self-Assembling Peptide Nanofiber Hydrogels in Tissue Engineering and Regenerative Medicine: Progress, Design Guidelines, and Applications: Self-Assembling Peptides in Tissue Engineering and Regeneration. J. Biomed. Mater. Res..

[34] Tibbitt M.W., Anseth K.S. (2009). Hydrogels as Extracellular Matrix Mimics for 3D Cell Culture. Biotechnol. Bioeng..

[35] Sieminski A.L., Semino C.E., Gong H., Kamm R.D. (2008). Primary Sequence of Ionic Self-Assembling Peptide Gels Affects Endothelial Cell Adhesion and Capillary Morphogenesis. J. Biomed. Mater. Res..

[36] Dou X.-Q., Feng C.-L. (2017). Amino Acids and Peptide-Based Supramolecular Hydrogels for Three-Dimensional Cell Culture. Adv. Mater..

[37] D’Auria G., Vacatello M., Falcigno L., Paduano L., Mangiapia G., Calvanese L., Gambaretto R., Dettin M., Paolillo L. (2009). Self-Assembling Properties of Ionic-Complementary Peptides: Self-Assembling Peptides. J. Pept. Sci..

[38] Gambaretto R., Tonin L., Di Bello C., Dettin M. (2008). Self-Assembling Peptides: Sequence, Secondary Structure in Solution and Film Formation. Biopolymers.

[39] Jung J.P., Jones J.L., Cronier S.A., Collier J.H. (2008). Modulating the Mechanical Properties of Self-Assembled Peptide Hydrogels via Native Chemical Ligation. Biomaterials.

[40] Zamuner A., Cavo M., Scaglione S., Messina G., Russo T., Gloria A., Marletta G., Dettin M. (2016). Design of Decorated Self-Assembling Peptide Hydrogels as Architecture for Mesenchymal Stem Cells. Materials.

[41] Conconi M.T., Ghezzo F., Dettin M., Urbani L., Grandi C., Guidolin D., Nico B., Di Bello C., Ribatti D., Parnigotto P.P. (2010). Effects on in Vitro and in Vivo Angiogenesis Induced by Small Peptides Carrying Adhesion Sequences. J. Pept. Sci..

[42] Ricci J.L., Clark E.A., Murriky A., Smay J.E. (2012). Three-Dimensional Printing of Bone Repair and Replacement Materials: Impact on Craniofacial Surgery. J. Craniofacial Surg..

[43] Castilho M., Moseke C., Ewald A., Gbureck U., Groll J., Pires I., Teßmar J., Vorndran E. (2014). Direct 3D Powder Printing of Biphasic Calcium Phosphate Scaffolds for Substitution of Complex Bone Defects. Biofabrication.

[44] Witek L., Shi Y., Smay J. (2017). Controlling Calcium and Phosphate Ion Release of 3D Printed Bioactive Ceramic Scaffolds: An in Vitro Study. J. Adv. Ceram..

[45] Isern J., Martín-Antonio B., Ghazanfari R., Martín A.M., López J.A., del Toro R., Sánchez-Aguilera A., Arranz L., Martín-Pérez D., Suárez-Lledó M. (2013). Self-Renewing Human Bone Marrow Mesenspheres Promote Hematopoietic Stem Cell Expansion. Cell Rep..

[46] Gharibi B., Cama G., Capurro M., Thompson I., Deb S., Di Silvio L., Hughes F.J. (2013). Gene Expression Responses to Mechanical Stimulation of Mesenchymal Stem Cells Seeded on Calcium Phosphate Cement. Tissue Eng. Part A.

[47] Gaetani M., Chinnici C.M., Carreca A.P., Di Pasquale C., Amico G., Conaldi P.G. (2018). Unbiased and Quantitative Proteomics Reveals Highly Increased Angiogenesis Induction by the Secretome of Mesenchymal Stromal Cells Isolated from Fetal Rather than Adult Skin. J. Tissue Eng. Regen. Med..

[48] Tasso R., Gaetani M., Molino E., Cattaneo A., Monticone M., Bachi A., Cancedda R. (2012). The Role of BFGF on the Ability of MSC to Activate Endogenous Regenerative Mechanisms in an Ectopic Bone Formation Model. Biomaterials.

[49] Thompson A., Schäfer J., Kuhn K., Kienle S., Schwarz J., Schmidt G., Neumann T., Hamon C. (2003). Tandem Mass Tags: A Novel Quantification Strategy for Comparative Analysis of Complex Protein Mixtures by MS/MS. Anal. Chem..

[50] Neto F., Klaus-Bergmann A., Ong Y.T., Alt S., Vion A.-C., Szymborska A., Carvalho J.R., Hollfinger I., Bartels-Klein E., Franco C.A. (2018). YAP and TAZ Regulate Adherens Junction Dynamics and Endothelial Cell Distribution during Vascular Development. eLife.

[51] Veschini L., Crippa L., Dondossola E., Doglioni C., Corti A., Ferrero E. (2011). The Vasostatin-1 Fragment of Chromogranin A Preserves a Quiescent Phenotype in Hypoxia-driven Endothelial Cells and Regulates Tumor Neovascularization. FASEB J..

[52] Veschini L., Belloni D., Foglieni C., Cangi M.G., Ferrarini M., Caligaris-Cappio F., Ferrero E. (2007). Hypoxia-Inducible Transcription Factor–1 Alpha Determines Sensitivity of Endothelial Cells to the Proteosome Inhibitor Bortezomib. Blood.

[53] Sweet L., Kang Y., Czisch C., Witek L., Shi Y., Smay J., Plant G.W., Yang Y. (2015). Geometrical versus Random β-TCP Scaffolds: Exploring the Effects on Schwann Cell Growth and Behavior. PLoS ONE.

[54] Diment L.E., Thompson M.S., Bergmann J.H.M. (2017). Clinical Efficacy and Effectiveness of 3D Printing: A Systematic Review. BMJ Open.

[55] Li X., Song T., Chen X., Wang M., Yang X., Xiao Y., Zhang X. (2019). Osteoinductivity of Porous Biphasic Calcium Phosphate Ceramic Spheres with Nanocrystalline and Their Efficacy in Guiding Bone Regeneration. ACS Appl. Mater. Interfaces.

[56] Fellah B.H., Josselin N., Chappard D., Weiss P., Layrolle P. (2007). Inflammatory Reaction in Rats Muscle after Implantation of Biphasic Calcium Phosphate Micro Particles. J. Mater. Sci. Mater. Med..

[57] Chen X., Wang J., Chen Y., Cai H., Yang X., Zhu X., Fan Y., Zhang X. (2016). Roles of Calcium Phosphate-Mediated Integrin Expression and MAPK Signaling Pathways in the Osteoblastic Differentiation of Mesenchymal Stem Cells. J. Mater. Chem. B.

[58] Piccolo S., Dupont S., Cordenonsi M. (2014). The Biology of YAP/TAZ: Hippo Signaling and Beyond. Physiol. Rev..

[59] Tavakol S., Mousavi S.M.M., Tavakol B., Hoveizi E., Ai J., Sorkhabadi S.M.R. (2017). Mechano-Transduction Signals Derived from Self-Assembling Peptide Nanofibers Containing Long Motif of Laminin Influence Neurogenesis in In-Vitro and In-Vivo. Mol. Neurobiol..

[60] Lutolf M.P., Gilbert P.M., Blau H.M. (2009). Designing Materials to Direct Stem-Cell Fate. Nature.

[61] Lutolf M.P., Hubbell J.A. (2005). Synthetic Biomaterials as Instructive Extracellular Microenvironments for Morphogenesis in Tissue Engineering. Nat. Biotechnol..

