# Supplementary material for: DAR 16-II Primes Endothelial Cells for Angiogenesis Improving Bone Ingrowth in 3D-Printed BCP Scaffolds and Regeneration of Critically Sized Bone Defects
Source: Biomolecules. 2022 Nov 2;12(11):1619. doi: 10.3390/biom12111619 (PMC9687468; doi:10.3390/biom12111619)
Supplement: Supplementary file 1 [file biomolecules-12-01619-s001.zip › biomolecules-1952263-Supplementary.pdf]

biomolecules

Supplementary Materials

DAR 16-II Primes Endothelial Cells for Angiogenesis Improving Bone Ingrowth in 3D-Printed BCP Scaffolds and Regeneration of Critically Sized Bone Defects

Eman Alfayez [1,2,†], Lorenzo Veschini [2,†], Monica Dettin [3], Annj Zamuner [4], Massimiliano Gaetani [5,6], Anna P. Carreca [5], Stevo Najman [7], Shahram Ghanaati [8], Trevor Coward [2] and Lucy Di Silvio [2,*]

[1] Faculty of Dentistry, Oral Biology Department, King Abdulaziz University, 21589 Jeddah, Saudi Arabia
[2] Faculty of Dentistry, Oral & Craniofacial Sciences King's College London, London SE1 9RT, UK
[3] Department of Industrial Engineering, University of Padua, 35131 Padua, Italy
[4] Department of Department of Civil, Environmental, and Architectural Engineering, University of Padua, 35131 Padua, Italy
[5] Fondazione Ricerca nel Mediterraneo (Ri.MED) and Department of Laboratory Medicine and Advanced Biotechnologies, Istituto di Ricovero e Cura a Carattere Scientifico-Istituto Mediterraneo per i Trapianti e Terapie ad Alta Specializzazione, 90100 Palermo, Italy
[6] Chemical Proteomics, Department of Medical Biochemistry and Biophysics, Karolinska Institutet and SciLifeLab (Science for Life Laboratory), SE-17 177 Stockholm, Sweden
[7] Faculty of Medicine, University of Niš, 18000 Niš, Serbia
[8] Department for Oral, Cranio-Maxillofacial and Facial Plastic Surgery, Medical Center of the Goethe University, 60323 Frankfurt, Germany
* Correspondence: lucy.di_silvio@kcl.ac.uk; Tel.: +02 07848 8475
† These authors equally contributed to this work.

Table S1. List of up-regulated proteins. Proteins increasing in amount in HUVEC cultured in the presence of DAR 16-II expressed as % increase over HUVEC cultured on gelatin. Only the proteins with highly statistically significant ($p < 0.01$, $n = 3$) differences are displayed in the table.

UniProt Accession	Gene ID	Average Amount Change %	Average deviation
P20839	IMPDH1	227.8	1.8
Q9H040	SPRTN	182.3	35.1
O95167	NDUFA3	170.6	8.3
Q9Y4Y9	LSM5	126.6	25.9
Q8TAK5	GABPB2	114.3	12.3
P02749	APOH	106.9	32.2
P02760	AMBP	106.4	4.3
Q86T82	USP37	102.2	35.3
P02787	TF	101.7	29.5
P01023	A2M	94.6	25.2
P23434	GCSH	82.6	13.4
Q9H8H2	DDX31	79.5	3.9
Q14520	HABP2	79.1	16.2
P02788	LTF	77.8	23.0
P02768	ALB	77.7	21.6
P60002	ELOF1	76.5	9.6
O75808	CAPN15	75.0	6.4
Q14191	WRN	71.4	14.3
P02462	COL4A1	68.1	1.1
Q8N357	SLC35F6	64.3	1.7
Q01484	ANK2	63.5	20.4
P35670	ATP7B	56.9	20.7
P04179	SOD2	56.1	5.3
Q9BQ15	NABP2	52.9	8.6
Q9Y6R0	NUMBL	51.7	2.8
P29353	SHC1	50.3	2.5
P02765	AHSG	49.1	18.6
Q9NZR1	TMOD2	48.6	7.8
Q86UY8	NT5DC3	46.2	3.2
P99999	CYCS	43.7	11.4
P14174	MIF	42.2	5.2
P51808	DYNLT3	42.1	2.4
P10253	GAA	40.9	6.7
Q9Y2Z9	COQ6	40.8	9.7
Q14687	GSE1	40.7	9.1
Q8IY63	AMOTL1	40.7	22.5
Q9UJF2	RASAL2	40.5	3.3
Q8IXK0	PHC2	40.1	3.0
O76024	WFS1	40.0	5.6

Table S2. List of down-regulated proteins. Proteins decreasing in amount in HUVEC cultured the presence of DAR 16-II expressed as % decrease over HUVEC cultured on gelatin. Only the proteins with highly statistically significant ($p < 0.01$, $n = 3$) differences are displayed in the table.

UniProt Accession	Gene ID	Average Amount Change %	Average deviation
P60673	PFN3	−40.1	8.4
Q96GC9	VMP1	−40.7	2.9
Q13796	SHROOM2	−41.0	0.8
Q86WR7	PROSER2	−41.1	0.9
Q8IZQ1	WDFY3	−41.3	1.9
Q13686	ALKBH1	−41.5	1.1
P28715	ERCC5	−42.3	0.6
Q96F10	SAT2	−43.3	2.3
P62857	RPS28	−43.4	0.8
Q969X6	CIRH1A	−43.7	1.4
Q96NS5	ASB16	−43.7	3.8
Q5T280	C9orf114	−44.3	0.8
Q9NQT4	EXOSC5	−44.3	1.2
Q8TE02	ELP5	−44.4	1.4
O00257	CBX4	−44.9	1.6
P10109	FDX1	−45.2	1.0
Q96R05	RBP7	−46.5	4.5
P41221	WNT5A	−46.6	3.7
Q92686	NRGN	−47.2	0.4
P42226	STAT6	−47.4	0.3
Q9H3C7	GGNBP2	−47.7	0.6
Q8TCT9	HM13	−48.5	1.2
Q2VPK5	CTU2	−48.6	1.7
Q8TBZ6	TRMT10A	−50.4	1.1
P43007	SLC1A4	−51.8	1.5
Q9Y4E5	ZNF451	−52.8	1.9
Q08722	CD47	−54.4	1.3
Q9NWQ8	PAG1	−55.8	6.0
Q6NYC1	JMJD6	−56.6	0.8
Q14697	GANAB	−59.1	0.3
Q8N475	FSTL5	−60.0	1.4
P09669	COX6C	−60.4	2.5
O96017	CHEK2	−61.0	3.3
075843	AP1G2	−62.0	2.6
Q9Y5J1	UTP18	−65.7	0.9
Q9NRR8	CDC42SE1	−66.2	2.5
